# The combined effect of dichloroacetate and 3-bromopyruvate on glucose metabolism in colorectal cancer cell line, HT-29; the mitochondrial pathway apoptosis

**DOI:** 10.1186/s12885-021-08564-3

**Published:** 2021-08-07

**Authors:** Hojatolla Nikravesh, Mohammad Javad Khodayar, Babak Behmanesh, Masoud Mahdavinia, Ali Teimoori, Soheila Alboghobeish, Leila Zeidooni

**Affiliations:** 1 Cellular and Molecular Research Center, Medical Basic Sciences Research Institute, Ahvaz Jundishapur University of Medical Sciences, Ahvaz, Iran; 2 Department of Toxicology, Faculty of Pharmacy, School of Pharmacy, Ahvaz Jundishapur University of Medical Sciences, Ahvaz, Iran; 3 Toxicology Research Center,Medical Basic Sciences Research Institute, Ahvaz Jundishapur University of Medical Sciences, Ahvaz, Iran; 4 Student Research Committee, Ahvaz Jundishapur University of Medical Sciences, Ahvaz, Iran; 5 Department of Virology, Faculty of Medicine, Hamadan University of Medical Sciences, Hamadan, Iran; 6 Department of Pharmacology, Faculty of Pharmacy, Ahvaz Jundishapur University of Medical Sciences, Ahvaz, Iran

**Keywords:** Apoptosis, 3-Bromopyruvate, Dichloroacetate, Colorectal cancer cells, 5-fluorouracil

## Abstract

**Background:**

5-Fluorouracil (5-FU) is regarded as the first line treatment for colorectal cancer; however, its effectiveness is limited by drug resistance. The ultimate goal of cancer therapy is induction of cancer cell death to achieve an effective outcome with minimal side effects. The present work aimed to assess the anti-cancer activities of mitocans which can be considered as an effective anticancer drug due to high specificity in targeting cancer cells.

**Methods:**

MTT (3–4,5-dimethylthiazol-2-yl-2,5-diphenyltetrazolium bromide) assay was performed to determine the effects of our mitocans on cell viability and cell death. Apoptosis and necrosis, caspase 3 activity, mitochondrial membrane potential and ROS production in HT29 cell lines were analyzed by ApopNexin™ FITC/PI Kit, Caspase- 3 Assay Kit, MitoTracker Green and DCFH-DA, respectively. Moreover, quantitative real-time polymerase chain reaction (qRT-PCR) was performed to detect the expression level of pro-apoptotic (Bax) and anti-apoptotic (Bcl-2) genes in HT29 cell lines.

**Results:**

Treatment with mitocans (3Br-P + DCA) inhibited the growth of HT29. Moreover, 3Br-P + DCA significantly induced apoptosis and necrosis, activation of caspase 3 activity, depolarize the mitochondrial membrane potential, and ROS production. At a molecular level, 3Br-P + DCA treatment remarkably down-regulated the expression of Bcl-2, while up-regulated the expression of Bax.

**Conclusion:**

Mitocans, in particular the combined drug, 3Br-P + DCA, could be regarded and more evaluated as a safe and effective compound for CRC treatment. Targeting hexokinase and pyruvate dehydrogenase kinase enzymes may be an option to overcome 5-FU -mediated chemo-resistant in colorectal cancer.

**Supplementary Information:**

The online version contains supplementary material available at 10.1186/s12885-021-08564-3.

## Background

Cancer, a pathology characterized by unchecked division of cells, is one of the fatal diseases with an increasing number of cases all over the world [1]. Malignant neoplasms with the source of the intestine, rectum and anal canal, classified as colorectal cancer (CRC), are the third and second leading causes of death in males and females in the world, respectively [2]. Approximately, up to 1.8 million new cases of CRC were reported to occur yearly in the world, with an estimated 881,000 cases dying of this malignancy [3]. Despite substantial progresses in CRC screening measures and treatment, the prognosis of patients with this cancer remains poor, demanding novel therapeutic strategies to manage the disease [4]. In this respect, 5-Fluorouracil (5-FU) is a well-known anti-cancer drug commonly used to treat CRC; the agent interferes with DNA and RNA to disrupt the metabolism of the uracil, which lead to the breakdown of DNA and apoptosis of the cancer cells. Nonetheless, it is reported that the resistance to 5-FU during the course of CRC treatment plays a major role in treatment failure [5–7]. Therefore, the expansion and development of new and more effective drug combinations for CRC treatment is guaranteed. Studies show that some anti-cancer agents, known as mitocans, which exert their anti-tumor activities by modulating mitochondria, can be considered as an effective anti-cancer drug due to high specificity in targeting cancer cells [8]. Dichloroacetate (DCA) and 3-bromopyruvate (3Br-P) are two such anti-tumor agents, which belong to class 7 (hexokinase inhibitors) and class 1,7 (Krebs cycle inhibitors, hexokinase inhibitors) of mitocans, respectively [8]. DCA activates pyruvate dehydrogenase by inhibiting the pyruvate dehydrogenase kinase enzyme, which ultimately boosts the production of Acetyl-CoA, as a precursor of the Krebs cycle. The enhancement of the Krebs’ cycle activity in turn increases the activity of the electron transfer chain; a function that increases the production of reactive oxygen species with mitochondrial origin, which lead to selective killing of cancer cells [9]. The another agent, 3Br-P, acts as a potent inhibitor of hexokinase; an enzyme that is overexpressed in cancer cells and is essential for the metabolism of cancer cells. The enzyme binds to voltage-dependent anion channel (VDAC, or porin) in the outer mitochondrial membrane, which results in resistance to chemotherapy by inhibiting glycolysis [10]. Recent studies reported that multi-target therapy acts more effective than single-target one [11]. Numerous studies have shown a synergistic action of DCA or 3Br-P with other compounds [12, 13]. However, the present work assessed the anticancer effects of 3Br-P in combined with DCA in a colorectal cancer cell line, HT-29.

## Methods

Human colorectal adenocarcinoma (HT-29) and human embryonic kidney 293 (HEK-293) cell lines were purchased from Iranian Biological Resource Center (Tehran, Iran). The anti-cancer agents, 5-FU and DCA/3Br-P, were supplied by Ebewe Pharma company and Sigma Aldrich (Saint Louis, MO), respectively. Dulbecco’s modified eagle’s medium (DMEM), fetal bovine serum (FBS), phosphate-buffered saline (PBS), and antibiotic (penicillin-streptomycin) were purchased from Invitrogen Co, (Carlsbad, CA). Furthermore, MTT (3-(4,5-dimethylthiazol-2-yl)-2, 5-diphenyl tetrazolium bromide), 2,7-dichlorofluorescein diacetate (DCFH-DA), Annexin V/FITC apoptosis detection kit and MitoTracker Green supplied by Sigma Aldrich (Saint Louis, MO) were applied in this work. Moreover, Caspase 3 Assay kit, Bax, Bcl-2, and GAPDH primers were obtained from Qiagen (Hilden, Germany).

### Cell culture and treatment

The cell lines, HT29 and HEK-293, were cultured in DMEM media with 10% FBS at 37 °C, under a humidified atmosphere with 5% CO2.

### Cell counting

The trypan blue exclusion dye assay was applied to determine the number of living cells in a cell suspension. Shortly, 20 μl of trypan blue dye 0.4% was added and mixed with equal parts of cell suspension in a micro tube. Subsequently, one side of a hemacytometer counter was filled with the cell suspension by placing the tip of the pipette at the notch, followed by counting the cells on the stage of a light microscope. The Calculation of cell percentage per milliliter was done via the following formula: cells/ml = average count per square × dilution factor × 10^4^. Moreover, the experiments were performed at least for three times [14].

### Cytotoxicity assays

Briefly, the HT-29 and HEK-293 cells were grown in 96-well plate (5000cells/well) in 100 μl medium and left overnight to adhere. Then, cells were treated with different concentrations of 3Br-P (40, 80, 160, and 320 μM); DCA (40, 80, 160 and 200 mM) and 5-FU (7.5, 15, 30, 60 and 120 μM) alone and combined with together, and incubate in media containing 10% FBS at 37 °C and 5–6.5% CO2 for 48 h. After treatment, 10 μl of the MTT (5 mg/ml in PBS) was added to each well and incubated for 3.5 h in 37 °C. Subsequently, the medium was removed and the formazan crystals were solubilized in 150 μl of DMSO. Plates were covered with foil and shacked on an orbital shaker for 15 min, and in the final step complete solubilization of the purple formazan crystals and reduced MTT were measured spectrophotometrically by microplate enzyme-linked immunosorbent assay (ELISA) reader at 570 nm according to the filters available for the ELISA reader. Viability inhibition was calculated as follows: cell viability (%) = Ab (test) /Ab (control) × 100 [15]. To calculate the Combination Index (CI), cells were treated with a combination of DCA and 3Br-P using the method of constant ratio drug combination proposed by Chou and Talalay [16].

### Measurement of apoptosis and necrosis

This was measured according to ApopNexin™ FITC/PI Kit instructions. In summary, 1.5 × 10^5^ cells were seeded and treated with different concentrations of 3Br-P, DCA and 5-FU alone and combined together in HT-29 cells for 48 h. After drug exposure, the cells were harvested, and then were washed with ice-cold PBS and stained with ApopNexin™ FITC kit for 15 min at room temperature in the dark. Stained samples were analyzed by Galaxy flow cytometer (ser. No: 0105362) [17].

### Caspase 3 assay

Caspase 3 activity was determined via using the specific caspase-3 substrate, prepared by Caspase- 3 Assay Kit, Colorimetric Qiagen (Hilden, Germany). In short, after treating the cells with 3Br-P, DCA and 5-FU alone and/or combined together, the caspase-3 activity was measured by ELISA at 504 nm, according to the protocol of the kit [18].

### Mitochondrial membrane potential (ΔΨm)

MitoTracker Green is a fluorescent color that selectively attaches covalently to mitochondrial proteins in the mitochondrial matrix by reacting with free thiol groups of cysteine roots. Briefly, in each well of 24-well plates, about 10^5^ cells were seeded and incubated overnight, and subsequently treated with 3Br-P, DCA and 5-FU alone and/or combined together for 48 h. Then, the cells were harvested and incubated with 10 μl of MitoTracker Green for 30 min at 37 °C in a 5% CO2 incubator. After incubation, the samples were placed on ice and immediately were assessed by flow cytometry [19].

### Determination of ROS production

To perform this, 24-well plates were seeded with 10^5^ cells in a complete culture medium and allowed to adhere overnight. In all wells (90% confluence) treated with 3Br-P, DCA and 5-FU alone and/or combined together, cells were washed twice with PBS, followed by staining with 10 μM DCFH-DA for 45 min. Then, after 2, 4, 8, and 12 h, the formation of fluorescent-oxidized DCF was measured by fluorimetry (535 nm excitation and 635 nm emission) [20].

#### RT-PCR and qPCR reaction

Total RNA was isolated according to the manufacturer’s instructions supplied by Rneasy Mini Kit (QIAgEN-cat. No. 74106.). Afterwards, cDNA was synthesized from 2 μg of the total RNA using the Prime Script RT-PCR kit (Quanti Tect Rev. Transcription Kit), according to the manufacturer’s instructions. Quantitative real-time PCR (qPCR) assays were performed with Roche LightCycler in 96-wells Gene Discs, using a final reaction volume of 20 μL containing 0.4 μl of each forward and reverse primers (with concentration of 10 μmol/L) (Table 1), 2x SYBR Green master mix (as indicated in the results section) and < 1 ng/μl of cDNA samples. The following thermal profile was applied; the cycling conditions used were as follows: 95 °C for 15 min; 30 cycles of 95 °C for 15 s, 66 °C for 30 Sec. Melting curve analysis was performed by ramping the *temperature* from 60 °C to 90 °C. All reactions were conducted in triplicate. The fold change of gene expression was calculated using 2^−ΔΔCt^ after normalizing to the expression level of GAPDH.

**Table 1:** Sequences of the primers and PCR product sizes used in RT-PCR

Gene	Primer	Sequence
**GAPDH**	**Sense**	**5′- CATCAATGGAAATCCCATCA − 3′**
	**Antisense**	**5′- GACTCCACGACGTACTCAGC − 3’**
**Bax**	**Sense**	**5′- GTCTTTTTCCGAGTGGCAGC − 3’**
	**Antisense**	**5′- GTCCAATGTCCAGCCCATGA − 3’**
**Bcl-2**	**Sense**	**5′- ATGTGTGTGGAGAGCGTCAA − 3’**
	**Antisense**	**5′- GGGCCGTACAGTTCCACAAA − 3’**

GAPDH, glyceraldehyde 3-phosphate dehydrogenase; Bax, Bcl-2-associated X protein; Bcl-2, B cell lymphoma-2

### Statistical analysis

All experiments were performed at least through 3 trials. All quantitative data are expressed as mean ± SD. One-way analysis of variance (ANOVA) followed by Tukey’s multiple comparisons were executed for comparison of different parameters between the groups using a GraphPad Prism 5 software (GraphPad Prism, San Diego, California, The U.S.A.).

## Results

### In vitro cytotoxicity

**Fig. 1:**
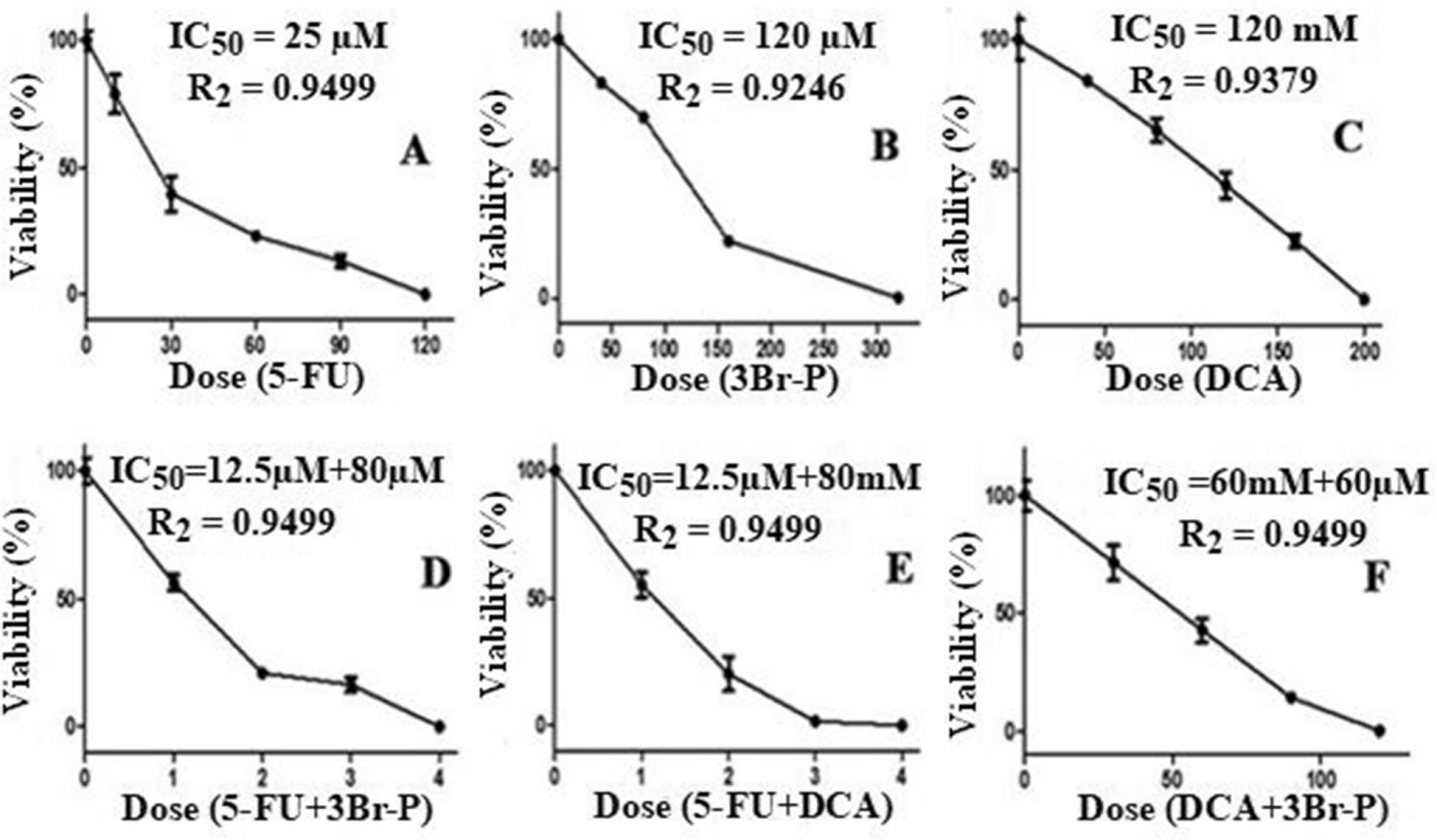
Cytotoxic activity of 3Br-P, DCA and 5-FU alone and combination together in colorectal cancer cell line (HT-29) for 48 h. Viability was assessed by the MTT reduction assay, as described in materials and methods. In D and E graphs, different doses of the our combination have been tested to assesse the viability, this combination on the horizontal axis shown by numbers

In the present study, cytotoxicity was assessed in the different cell lines by using MTT assay. The IC50 values for the HT-29 cell line obtained from the study are reported as follows: 5-FU;25 μM, 3Br-P;120 μM, DCA;120 mM, 5-FU + 3Br-P;12.5 μM + 80 μM, 5-FU + DCA;12.5 μM + 80 mM, 3Br-P + DCA; 60 μM + 60 mM. Nonetheless, in the normal cell line, HEK-293, the above mentioned doses had no significant effects at 48 h after treatment (Fig. 1). The data with HEK-293 cells shown in additional results.

### In vitro ROS activity

**Fig. 2:**
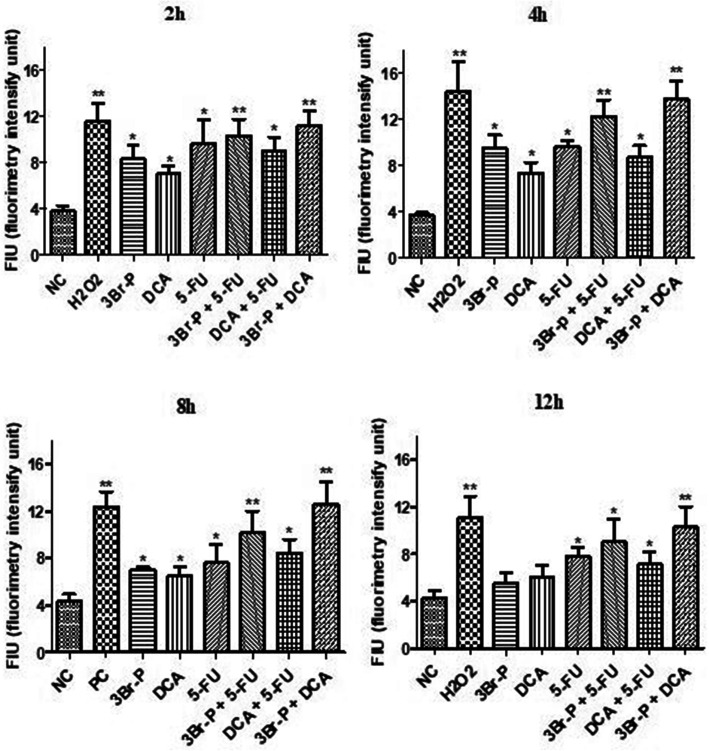
Effect of 3Br-P, DCA, and 5-FU alone and/or combined together on ROS production in HT-29 cell line after 2 h, 4 h, 8 h, and 12 h. Data presented as Mean ± SEM. *: (*p*<0.05) designates significant difference with control, ^#^: (*p<0*.05) designates significant difference with control, 3Br-P, DCA, 5-FU alone and/or combined together

The ROS activity in the treated group was shown to be significantly higher than the untreated ones, at the times of 2, 4 and 8 h after treatment. Furthermore, among the mentioned times in the treated group, the ROS amount was found to be higher during 4 h after treatment. Moreover, the combinations of 5-FU + 3Br-P and 3Br-P + DCA in the treated HT-29 cells produced more ROS amount than the other compounds (Fig. 2).

### In vitro assessment of mitochondrial membrane potential (ΔΨm)

**Fig. 3:**
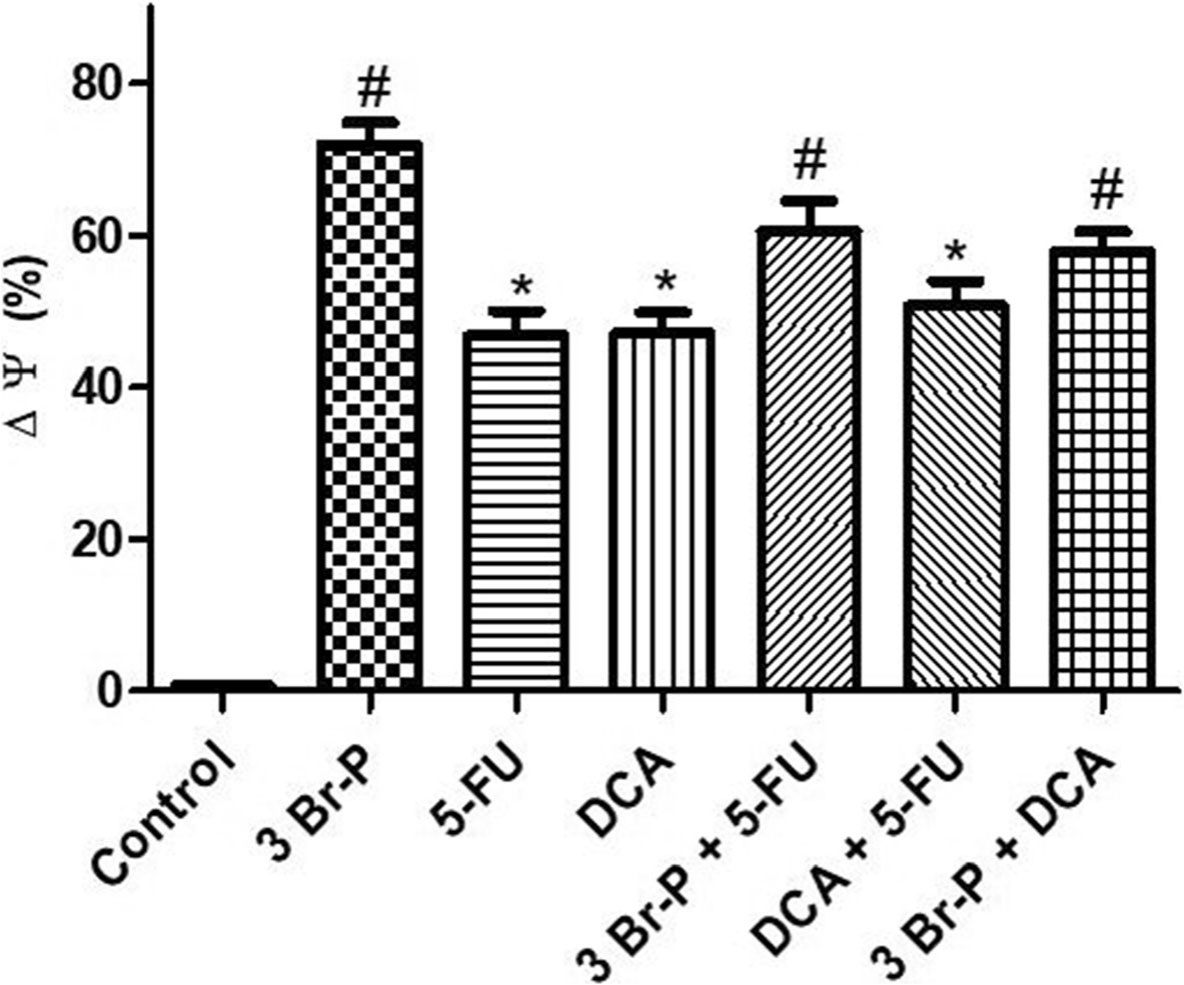
Effect of 3Br-P, DCA, and 5-FU alone and/or combined together on loss of mitochondrial membrane potential (Δψ) in HT-29 cell line after 48 h. Data presented as Mean ± SEM. *: (*p*<0.05) designates significant difference with control, ^#^: (*p*<0.05) designates significant difference with control, 5-FU, DCA and DCA + 5-FU

Based on our results, the compound 3Br-P significantly promoted ΔΨm reduction in HT-29 cells in comparison with the control and other treated groups. These results show that the combined agents, including 5-FU + 3Br-P, 5-FU+ DCA, and 3Br-P + DCA significantly increased ΔΨm lose in HT-29 cells in compared to the control (Fig. 3).

**Fig. 4:**
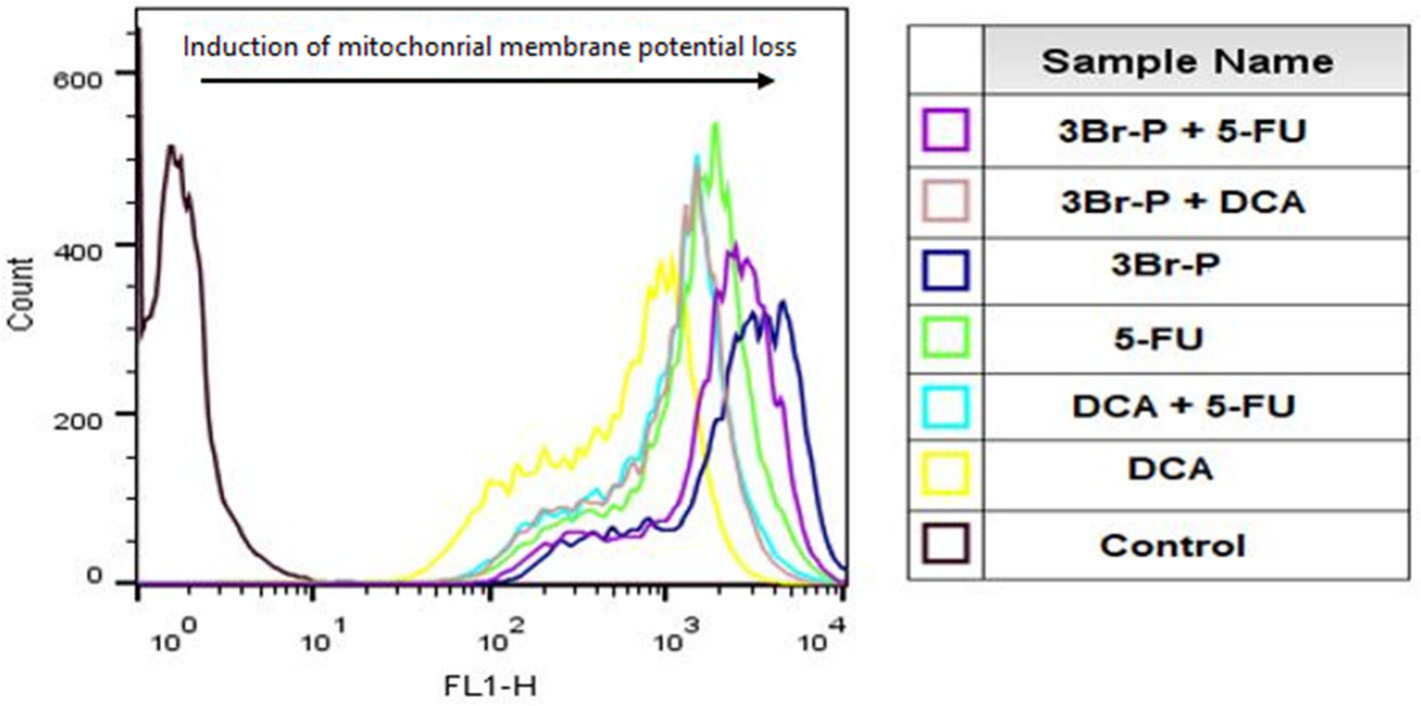
Mitochondrial membrane potential was measured using fluorescent dye mito tracker green and flow cytometry in colorectal cancer cell line (HT-29) treated with 3Br-P, DCA and 5-FU alone and/or combined together. (FL1 525 nm band pass filter green)

**Fig. 5:**
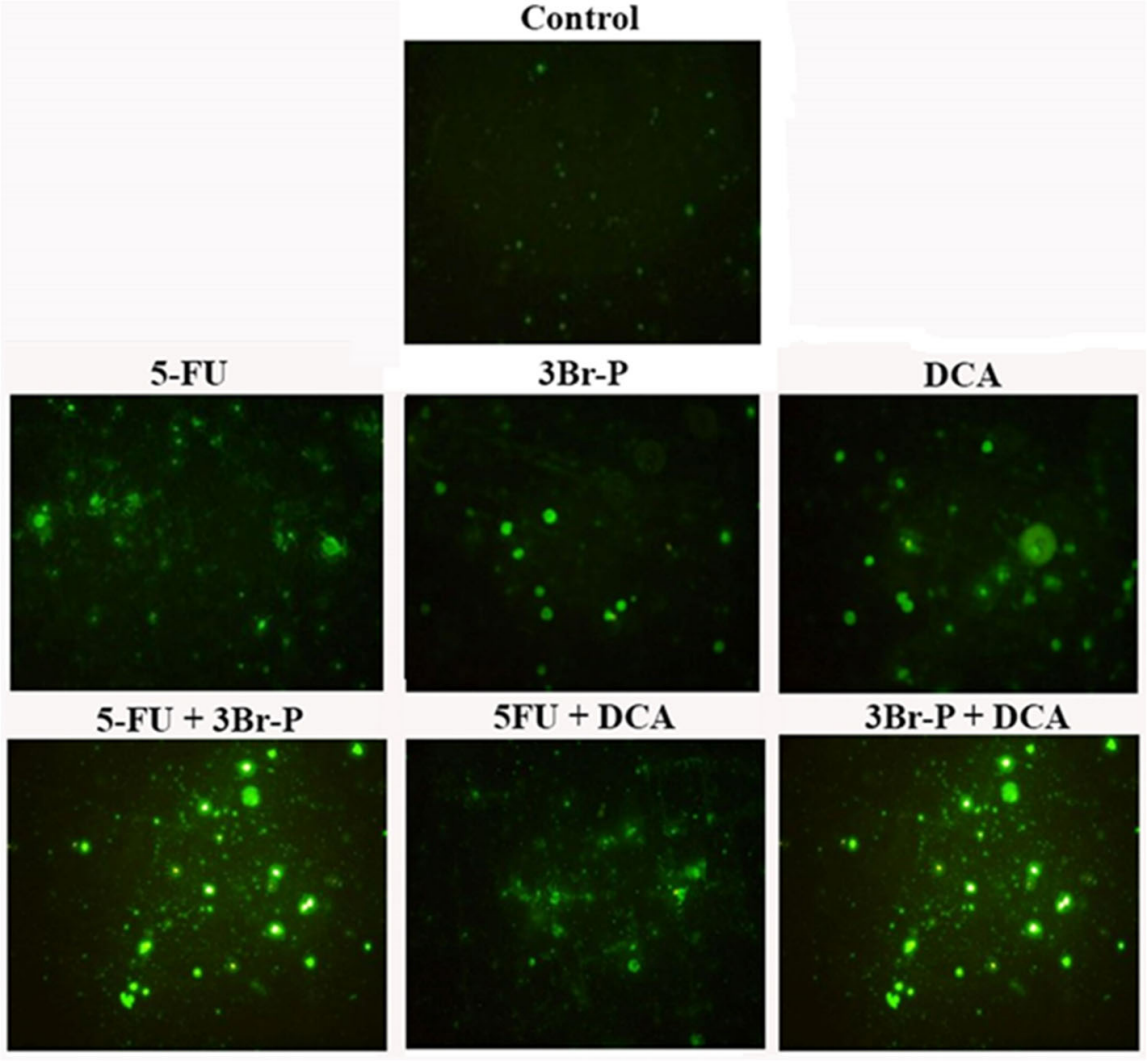
Mitochondrial membrane potential changes in colorectal cancer cell line (HT-29) measured via a fluorescence microscope (20X) 48 h after exposure to 3Br-P, DCA, and 5-FU alone and/or combined together

Mitotracker green dye accumulates in the mitochondrial matrix where it covalently reacts with free thiol groups of cysteine residues and produce green fluorescence (FL1 525 nm band pass filter green). The intensity of this emitted fluorescent indicates mitochondrial membrane potential loss (Fig. 4). Changes in mitochondrial morphology and mitochondrial fragmentation measured via a fluorescence microscope, the intensity of this emitted fluorescent also indicates the mitochondrial degeneration (Fig. 5).

### Caspase 3 assay

**Fig. 6:**
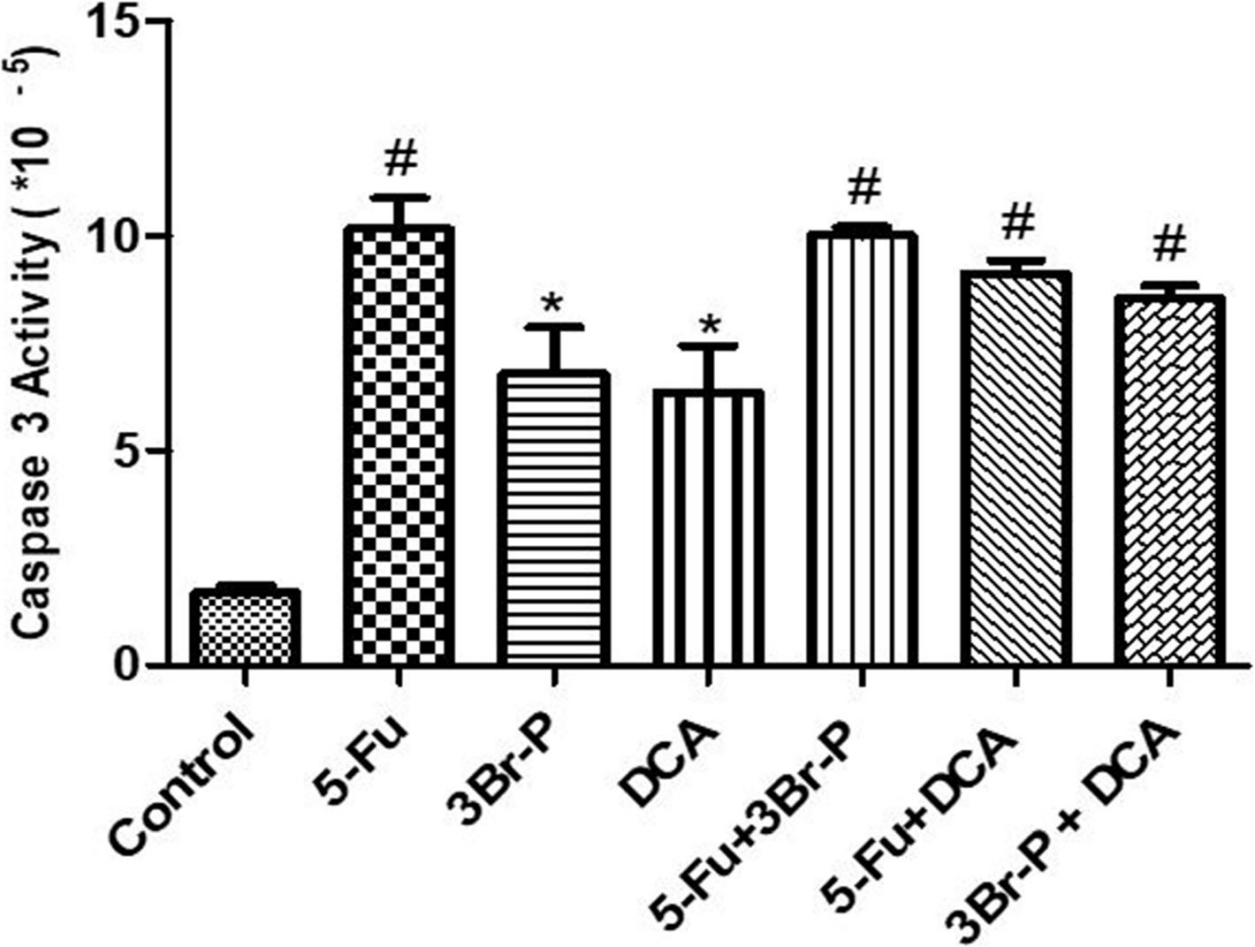
Effect of 3Br-P, DCA, and 5-FU alone and/or combined together on caspase 3 activity in HT-29 cell line after 48 h. Data presented as Mean ± SEM. *: (*p*<0.05) designates significant difference with control. ^#^: (*p*<0.05) designates significant difference with control, DCA and 3Br-P

Treatment of the HT-29 cells with all the assessed compounds (5-FU, 3Br-P, DCA, 5-FU+ DCA, 5-FU + 3Br-P, and 3Br-P + DCA) significantly increased the caspase 3 activity in compared to untreated cells (*p* ≤ 0.05) (Fig. 6).

### Quantification of the Bax/Bcl-2 ratio

**Fig. 7:**
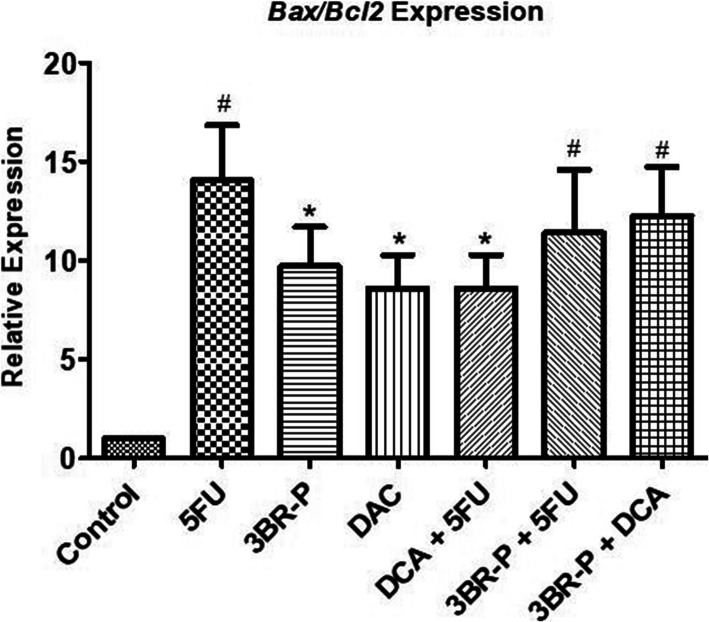
Effect of 3Br-P, DCA, and 5-FU alone and/or combined together on gene expression, in HT-29 cell line after 48 h. Data presented as Mean ± SEM. *:(*p*<0.05) designates different with control, ^#^:(*p*<0.05) designates different with control, 3Br-P, DCA, 5-FU + DCA

Treatment of the HT-29 cells with 5-FU, 3Br-P, DCA, 5-FU + DCA, 5-FU + 3Br-P, and 3Br-P + DCA significantly increased the Bax/Bcl-2 genes expression in compared to the control ones (*p* ≤ 0.05). Interestingly, there was no a significant difference of the Bax/Bcl-2 gene expression between HT-29 cells treated with 5-FU alone and those treated by 3Br-P + DCA (Fig. 7).

### Induction of apoptosis and necrosis by flow cytometry

**Fig. 8:**
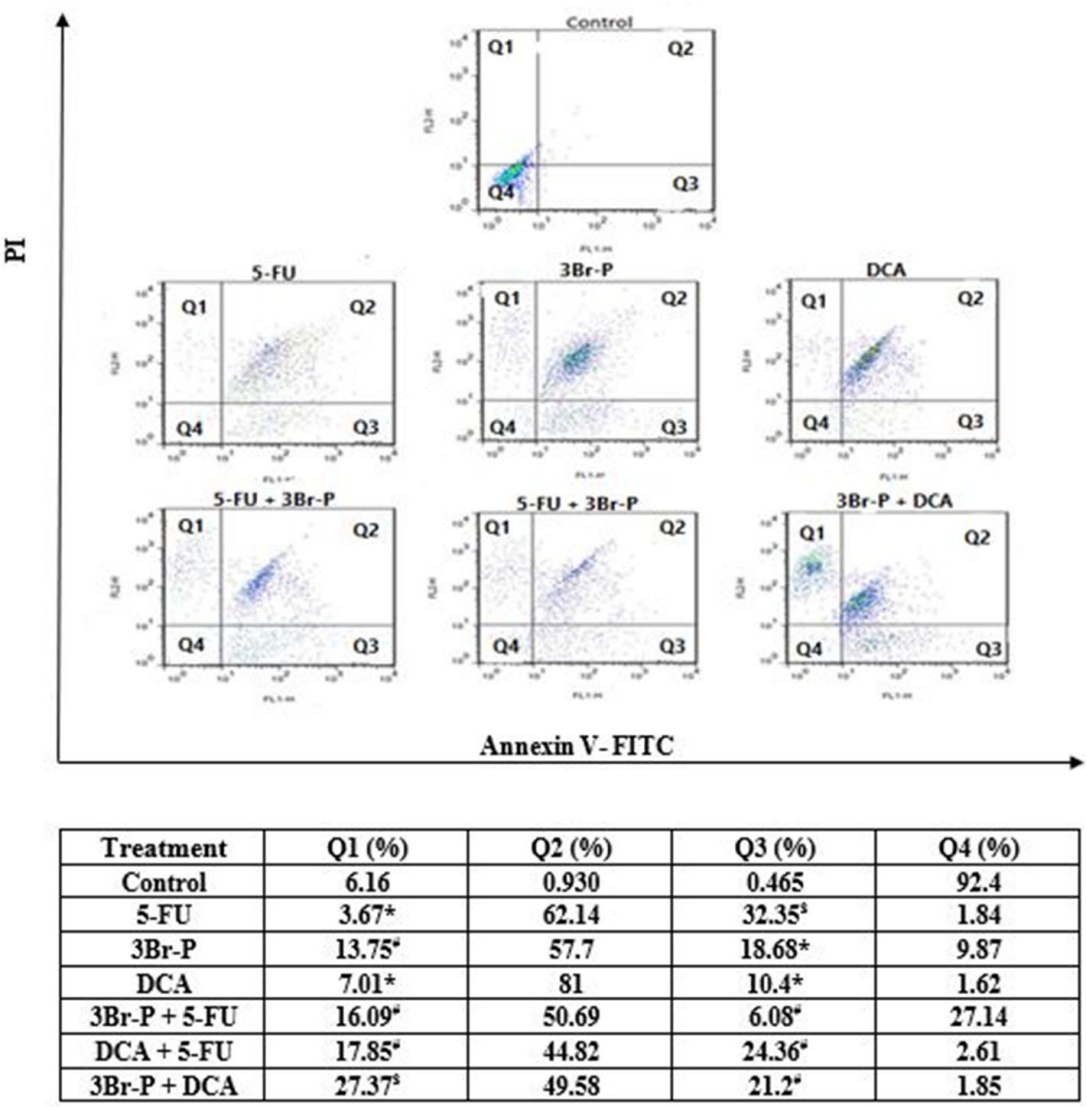
Fig. 8: Flow cytometry assessment of apoptosis and necrosis in HT-29 cell line after 48 h treatment with 3Br-P, DCA, and 5-FU alone and/or combined together. Data presented as Mean ± SEM. *: (*p*<0.05) designates significant difference with control, #: (*p*<0.05) designates significant difference with control, 3 Br-P and DCA, ^$^: (*p*<0.05) designates significant difference with control, 3Br-P, DCA, and/or combined together. Treated cells were stained with Annexin V-FITC/ PI assay. Four quadrants (Q) representing normal cells (Q4), early apoptosis cells (Q3), late apoptotic/necrotic cells (Q2) and necrotic cells (Q1)

Annexin-V FITC/PI staining confirmed that 5-FU, 3Br-P, DCA, 5-FU + DCA, 5-FU + 3Br-P, and 3Br-P + DCA could induce apoptosis in HT-29 cells. All the treated HT-29 cells had a significant higher necrotic pattern as compared to untreated cells (p ≤ 0.05). It was found that the treatment with 5-FU significantly increases the percentage of apoptosis from 0.465% in the control cells to 32.35% in the treating cells. Moreover, the 5-FU + DCA, 5-FU + 3Br-P, and 3Br-P + DCA groups had a significantly increased apoptosis in compared to the control, 3Br-P and DCA groups (Fig. 8).

## Discussion

**Fig. 9:**
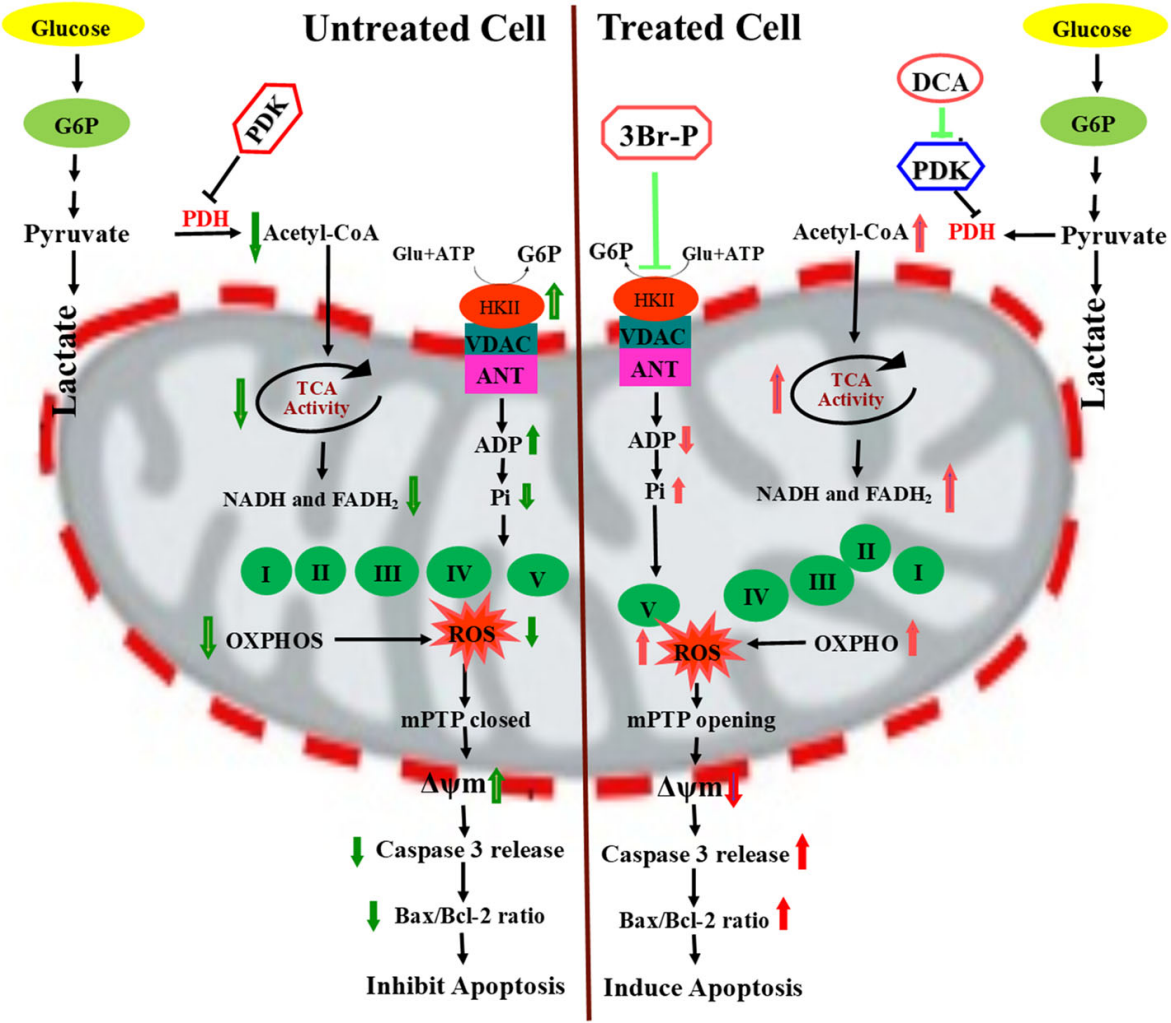
Represented regarding apoptotic effects of 3Br-P combined with DCA in colorectal cancer cell line HT-29

Globally, CRC is the second and third leading causes of death in women and men, respectively [21]. The aim of cancer therapy is to inhibit proliferation (cytostatic effects) and to induce cell death (cytotoxic effects) of cancer cells [22], to achieve an effective outcome with minimal side effects [23]. In most cancers, the first therapeutic strategy used to treat is surgical removal. Obviously, surgery for some types of cancers is relatively simple and for other types is impractical [23]. Therefore, chemotherapy and radiotherapy are used to control or eradicate metastatic cells. Traditional chemotherapy is the use of drugs to target the cell cycle, DNA, RNA, and proteins in cancer cells. 5-FU is the first traditional chemotherapy and the first choice for CRC therapy, whose effectiveness is limited by drug resistance [24]. Mitochondria have emerged as an attractive target for anticancer drugs, so that the compounds such as mitocans that target mitochondria with anti-cancer activity has become a focus of recent research due to their great clinical potential [24, 25]. In our study, we have described the cytotoxic effect of the mitochondria-targeted drugs, 3Br-P + DCA, in HT-29 cell line and assessed the mechanism of its action. Recent studies have shown that therapies having multiple targets result in greater benefits than single-targeted therapies [26]. There are several studies suggesting a synergistic action of 3Br-P or DCA with other compounds. For instance, the selective inhibitor of glycolytic enzymes, 3Br-P, when combined with sodium citrate (SCT), resulted in reduced ATP level, induced mitochondrial-mediated apoptosis, and suppressed tumor growth in gastric cancer cells [27]. In the same way, when cisplatin or oxaliplatin combined with 3Br-P, amazingly boosted the antiproliferative effects of the platinum drugs in HCT116 cells [28]. Moreover, antitumor effects of tamoxifen, the first-line adjuvant endocrine therapy for estrogen receptor-positive breast cancer, were markedly improved when used with 3Br-P, as a sensitizer to target glycolysis [29]. On the other hand, in colorectal cancer cells parallel results were obtained using traditional chemotherapy drugs with DCA. Ayyanathan et al. demonstrated that the combination of DCA and sulindac augments the selective killing of A549 and SCC25 cancer cells via ROS production, mitochondrial dysfunction, and apoptosis [30]. Similarly, Florio et al., reported that the combination of three metabolic drugs, DCA, GW6471, and metformin resulted in a remarkable enhanced ROS production, modulated cell cycle progression, and promoted apoptosis in PGL cells [31]. These findings are in line with the results reported in our study. In the present study, we found that DCA and 3Br-P together or in combined with 5-FU significantly reduced cell viability and proliferation of HT-29 cell lines (Fig. 8). Apoptosis plays an important role in treatment of cancer, as it is a popular target of many treatment strategies [32]. Accordingly, we showed that the combined drug therapy, DCA + 3Br-P, promotes apoptosis more effectively than the single therapies in HT-29 cell lines (Fig. 8); a mechanism that may be induced by modulating pathways such as ROS-dependent apoptosis in HT-29 cell lines. In the mitochondrial (caspase-dependent intrinsic) pathway that induces apoptosis, caspases, in particular caspase-3, are crucial mediators for the apoptotic machinery in many cell types [33]. This pathway is controlled by B-cell lymphoma-2 (BCL2) family proteins, composed of members that either promote or inhibit apoptosis. A major checkpoint in cellular fate is a balance formed between pro-apoptotic (Bax) and anti-apoptotic (Bcl-2) members [34]. Increased Bax/Bcl-2 ratio up-regulates caspase-3, which leads to apoptosis of tumor cells [35]. In the current study, we evaluated caspase 3 release, Bax/Bcl-2 ratio expression and mitochondrial membrane potential (ΔΨm). The results demonstrated that the three mentioned parameters increased by the combined drug therapy (DCA + 3Br-P) in comparison to single drug therapy. As a result, more studies are needed to investigate the mechanisms underlying the anticancer effect of combined 3Br-P + DCA and a combination of other mitocans treatment on CRC cells (Fig. 9).

## Conclusion

We have indicated that a combination of 3Br-P + DCA exerts an antitumor effect in vitro by activation of mitochondria-dependent apoptosis. The first goal of target therapy is to fight cancer cells with more precision and fewer side effects potentially. Targeted cancer therapies were designed to target a specific molecule that is critical for the growth and expansion of cancer cell, hence, blocking these molecules can kill cancer cells without any harm to normal cells. According to the mitocans features, we can consider them as apoptosis inducers. However, the results of the combination of 3Br-P + DCA in this study have no significant difference with the 5-FU alone; however, we should never ignore the primary goal of this study to show that combination therapy has lower side effects and resistance to treatment.

## Supplementary Information

**Additional file 1.**

## Data Availability

The datasets used and/or analyzed during the current study are available from the corresponding author on reasonable request.

## Abbreviations

CRC: colorectal cancer
5-FU: 5-Fluorouracil
MTT: 3–4,5-dimethylthiazol-2-yl-2,5-diphenyltetrazolium bromide
qRT-PCR: quantitative real-time polymerase chain reaction
DCA: Dichloroacetate
3Br-P: 3-bromopyruvate
VDAC: voltage-dependent anion channel
HT-29: Human colorectal adenocarcinoma
HEK-293: human embryonic kidney 293
SCT: sodium citrate
