# Supplementary material for: The combined effect of dichloroacetate and 3-bromopyruvate on glucose metabolism in colorectal cancer cell line, HT-29; the mitochondrial pathway apoptosis
Source: BMC Cancer. 2021 Aug 7;21:903. doi: 10.1186/s12885-021-08564-3 (PMC8349486; doi:10.1186/s12885-021-08564-3)
Supplement: Supplementary file 1 — Additional file 1. [file 12885_2021_8564_MOESM1_ESM.docx]

Cytotoxic activity of 3Br-P (120 µM), DCA (120 mM) and 5-FU (25 µM) alone and combination DCA + 5-FU, 3Br-P + 5-FU and 3Br-P + DCA together in HEK 293 cell line for 48h. Viability was assessed by the MTT reduction assay, as described in materials and methods.
